# Biologically Inspired Medical Multi-Modal Dataset Distillation via Contrast-Aware Alignment and Memory Compression

**DOI:** 10.3390/biomimetics11050314

**Published:** 2026-05-02

**Authors:** Taoli Du, Ziming Wang, Yue Wang, Ming Ma, Wenhui Li

**Affiliations:** 1 College of Computer Science and Technology, Jilin University, Changchun 130012, China; dutl21@mails.jlu.edu.cn (T.D.); ziming22@mails.jlu.edu.cn (Z.W.); yue_wang20@mails.jlu.edu.cn (Y.W.); maming22@mails.jlu.edu.cn (M.M.); 2 School of Games, Jilin Animation Institute, Changchun 130013, China

**Keywords:** dataset distillation, biological vision system, bio-inspired mechanism, contrastive learning, multi-modal medical imaging

## Abstract

Multi-modal Magnetic Resonance Imaging (MRI) provides complementary information for clinical diagnosis, yet its large-scale storage, privacy sensitivity, and annotation cost pose significant challenges. Inspired by biological vision systems, which integrate multi-sensory inputs and compress experiences into compact memory representations, we propose a bio-inspired framework termed Contrast-Guided Multi-modal Dataset Distillation (CGMDD). In biological perception, different sensory channels observe the same environment from complementary perspectives, while hierarchical neural processing ensures perceptual consistency across modalities. Meanwhile, memory systems such as the associated medial temporal lobe structures consolidate redundant experiences into efficient representations for long-term storage. Motivated by these principles, CGMDD treats multi-modal MRI as multi-view perceptual signals and introduces a hierarchical cross-modal contrastive learning mechanism that enforces perceptual alignment across modalities, analogous to multi-level processing in the visual cortex. Furthermore, we design a dynamic dataset distillation strategy that mimics memory consolidation by compressing large-scale data into compact, informative synthetic representations through gradient-based optimization. The proposed framework jointly optimizes perceptual alignment and memory compression in an end-to-end manner, achieving a biologically plausible integration of perception and learning. Experimental results on two MRI datasets demonstrate that CGMDD can compress the original dataset to 5% of its size while maintaining competitive performance, even with only 30% of the labels. These findings highlight the effectiveness of bio-inspired mechanisms in building efficient, robust, and privacy-preserving computer vision systems.

## 1. Introduction

Biological vision systems exhibit remarkable efficiency, robustness, and adaptability when processing complex visual environments [1,2]. Unlike conventional artificial vision systems that often rely on large-scale data and heavily engineered pipelines, biological vision operates through the integration of multiple visual cues and the progressive compression of redundant information into compact and efficient representations. This dual capability—multi-view visual integration and efficient representation formation—enables reliable perception even under limited computational resources and incomplete observations, providing an important paradigm for the design of intelligent vision systems.

In medical imaging, multi-modal Magnetic Resonance Imaging (MRI) can be naturally interpreted as an artificial counterpart to multi-view visual perception. Different imaging modalities (e.g., T1, T2, FLAIR) capture complementary anatomical and functional characteristics of the same underlying structures, analogous to how the visual system integrates diverse visual inputs across viewpoints and feature scales. However, the large-scale nature of multi-modal MRI data introduces significant challenges, including high storage and computational costs [3,4], stringent privacy requirements [5,6,7], and the limited availability of high-quality annotations, all of which hinder its broader deployment and utilization.

Dataset distillation has emerged as a promising approach to address these challenges by compressing large datasets into compact synthetic representations with high information density [8]. Despite recent progress, most existing methods are primarily designed for unimodal natural images [9,10] and fail to fully exploit the intrinsic relationships among multiple modalities. In particular, they lack mechanisms to enforce consistency across views of the same underlying structure, leading to the loss of complementary information during the distillation process. This limitation becomes especially critical in medical imaging, where subtle structural and functional cues are essential for accurate diagnosis.

Existing dataset distillation research has established several representative optimization paradigms, including gradient matching, distribution matching, and trajectory matching. In particular, dataset condensation with gradient matching [11] demonstrated that synthetic samples can be optimized by aligning the gradients produced by real and distilled data, while dataset meta-learning from kernel ridge-regression [12] provided a complementary perspective by formulating dataset distillation through kernel-based meta-learning. These studies have laid the methodological foundation for data-efficient learning, but they were primarily developed for unimodal natural image benchmarks and do not explicitly account for the cross-modal correspondence and complementary semantics inherent in medical multi-modal imaging.

From a biological perspective, perception and memory representation formation are tightly coupled processes within the visual system. The visual cortex performs hierarchical feature extraction, progressively integrating low-level and high-level information from multiple visual inputs, and aligning information across visual channels to form coherent representations [13]. Meanwhile, the memory system reduces redundant visual information through efficient coding mechanisms, consolidating experiences into compact yet informative memory representations. Inspired by these principles, we reinterpret multi-modal dataset distillation as a unified process of multi-view perceptual alignment and memory compression.

In the medical domain, recent studies have begun to explore data-efficient learning and compact data representation for imaging tasks, particularly in single-modality settings where distilled or condensed datasets are used to alleviate annotation cost and data sharing burdens [14,15,16,17]. Meanwhile, multi-modal medical image analysis has made substantial progress through early fusion, late fusion, and intermediate fusion strategiess [18], as well as cross-modal contrastive learning [19,20], showing the importance of exploiting complementary information across modalities. Nevertheless, these two lines of research remain largely disconnected: existing medical image distillation methods rarely model modality-level consistency explicitly, whereas multi-modal medical learning methods typically assume access to the full dataset rather than its highly compact distilled counterpart.

Therefore, a principled framework is needed to bridge the gap between dataset distillation and multi-modal medical representation learning. To address this limitation, we propose a Contrast-Guided Multi-modal Dataset Distillation (CGMDD) framework that integrates these bio-inspired mechanisms [21] into a unified learning paradigm. In contrast to existing methods, CGMDD is designed to jointly model cross-modal alignment and dataset compression, rather than treating them as separate problems. Specifically, we design a modality-aware feature encoding strategy that captures both shared and modality-specific representations, analogous to multi-level processing in the visual cortex. Building upon this representation, we introduce a hierarchical cross-modal contrastive learning strategy [22] to enforce consistency across modalities, mimicking how the visual system aligns multiple visual observations into a coherent percept. In parallel, we develop a dynamic dataset distillation mechanism that mimics memory consolidation by compressing large-scale data into compact synthetic representations through gradient matching, reflecting the efficient coding principle in biological vision.

**Figure 1:**
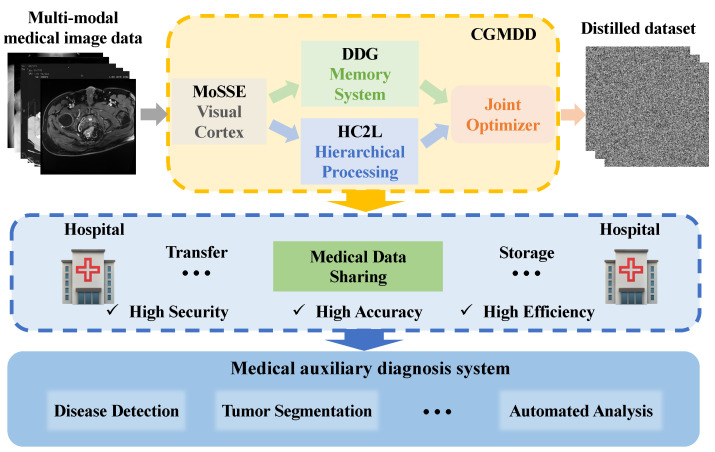
Graphical overview of the proposed CGMDD framework for efficient and privacy-aware medical data sharing.

By jointly optimizing these processes, CGMDD achieves a biologically inspired balance between representation learning and data compression. The resulting system not only preserves critical diagnostic information but also enables efficient and privacy-preserving medical data sharing under resource-constrained scenarios. The key novelty lies in the coordinated integration of contrastive learning and dataset distillation under a multi-modal setting, enabling effective compression while preserving cross-modal consistency. Extensive experiments demonstrate that the proposed method maintains strong performance even under extreme compression ratios and limited annotation settings. To our knowledge, this is the first attempt to jointly optimize contrastive learning and dataset distillation for multi-modal medical imaging, achieving the synergistic optimization of the “compression–performance–privacy” triad. A graphical overview of the proposed framework is shown in Figure 1.

Our contributions are summarized as follows:

- We propose a biologically inspired framework that unifies multi-modal perception alignment and memory compression for dataset distillation.
- We design a hierarchical cross-modal contrastive mechanism that mimics perceptual alignment in the visual cortex.
- We introduce a dynamic distillation strategy inspired by memory consolidation processes in biological systems.
- Extensive experiments demonstrate that the proposed method achieves strong performance under extreme compression and limited annotation settings.

## 2. Related Work

### 2.1. Dataset Distillation and Condensation

Dataset distillation, also known as dataset condensation, is an emerging paradigm for data-efficient learning, inspired by network distillation [23] and model compression [24]. The concept was formally introduced by [25], with the core objective of synthesizing a compact yet information-rich dataset such that models trained on it achieve comparable performance to those trained on the full, original dataset.

Early research mainly focused on coreset selection [26], where the most representative subset of real data is selected for training. In recent years, however, mainstream methods have shifted toward directly optimizing synthetic data via parameterized approaches, including gradient matching [11], distribution matching [10], and trajectory matching [27]. Gradient matching minimizes the discrepancy between gradients computed on synthetic and real data to enable knowledge transfer. For instance, the DC [11] method introduced this idea, while IDC [28] improved it by storing synthetic data at lower resolution. MTT [27] matches parameters after multiple training steps, essentially performing long-term gradient matching. RCIG [29] leverages implicit gradients to compute meta-gradients, and TESLA [30] reduces memory overhead in MTT. Shin et al. [31] matched the sharpness of losses between real and synthetic data, which is akin to gradient matching. Distribution matching focuses on aligning the distribution moments of real and synthetic data in the feature space. Following the initial DD work [25], KIP [12] incorporated ridge regression to reduce computational cost and extended the approach to infinitely wide networks [32]. RFAD [33] replaces the kernel in KIP with a neural network Gaussian process kernel. DM [10] uses a class encoder to extract features and aligns distribution between real and synthetic datasets for each class. IDM [34] further enhances DM by introducing regularization and model queues. Trajectory matching aims to replicate the parameter update trajectory during training, fundamentally similar to gradient matching. FTD [35] proposes to regularize the flatness of multiple trajectories to reduce error accumulation and enhance network robustness. DATM [36] finds that the choice of trajectory phase (early or late) significantly impacts performance, suggesting the use of early-stage trajectories for low images per class (IPC) and later stages for high IPC. SeqMatch [37] identifies challenges in learning high-level features in high-IPC scenarios and proposes partitioning the synthetic dataset into subsets, each derived from different phases of training trajectories.

While existing methods have demonstrated strong performance on natural image tasks, they still face significant challenges when applied to multi-modal medical imaging. First, the lack of cross-modal coordination limits their ability to capture complementary relationships and maintain anatomical consistency across modalities, especially when directly extending single-modal approaches via simple concatenation. Second, their focus on low-level pixel features makes it difficult to preserve subtle yet clinically critical lesion information, such as tumor boundary infiltration and small-scale abnormalities.

### 2.2. Efficient Learning for Multi-Modal Medical Imaging

Multi-modal fusion is a critical approach to enhancing diagnostic accuracy. Current mainstream strategies include early fusion (concatenating or weighting multi-modal data at the input stage) [19,38], late fusion (training individual modality-specific models independently and integrating them at the decision level) [39,40,41], and intermediate fusion (performing feature interaction and integration at various network layers) [18,42], such as incorporating attention mechanisms [20], graph neural networks [43], or Transformer architectures [44,45]. Recently, cross-modal contrastive learning [46,47] has been widely applied to representation learning in multi-modal medical imaging. By pulling representations of different modalities from the same instance closer together on the hypersphere (i.e., shared feature space) and simultaneously pushing representations of different instances further apart, it effectively learns modality-invariant features and improves the model’s robustness to modality missingness or noise.

However, existing multi-modal learning methods largely rely on full training datasets and thus fail to address the storage and computational burden of large-scale multi-modal data at the source. Although cross-modal knowledge distillation has been explored, it mainly operates at the model level and is not designed for data-level compression or reconstruction, leaving the integration of contrastive alignment and dataset distillation within a unified optimization framework largely underexplored.

### 2.3. Medical Data Sharing and Privacy Protection

To address the challenges of data scarcity and privacy sensitivity in medical imaging, researchers have proposed a variety of solutions, including data augmentation (e.g., random transformations and generative models such as GANs or diffusion models) [48,49,50,51], transfer learning (fine-tuning pre-trained models) [52], lightweight network design (e.g., 3D variants of MobileNet and ShuffleNet) [53], and federated learning (privacy-preserving distributed training). While effective in specific contexts, these methods do not fundamentally reduce reliance on the original datasets. Data augmentation emphasizes data expansion rather than compressing [54,55]; transfer learning still depends on real samples from the target task for fine-tuning; lightweight models reduce computational overhead often at the expense of performance; and federated learning, despite its data privacy benefits, still requires complete data support locally. In contrast, dataset distillation offers a fundamentally different solution by directly compressing the data itself. It significantly reduces the cost of computation, storage, and transmission while maintaining competitive performance. Unlike the method of removing identifiers to protect patient privacy [56], or the privacy-preserving medical data sharing schemes in cloud environments proposed by Yang et al. [57] and Fabian et al. [58], which only achieve superficial anonymization, dataset distillation eliminates potential privacy leakage risks at the data generation level. It enables genuine, complete anonymization of medical image data itself, thereby fundamentally addressing privacy protection—the primary barrier to secure medical data sharing [59].

Our work, in response to the aforementioned limitations and inspired by the hierarchical perception and multi-view integration mechanisms of the biological vision system, proposes a unified framework that integrates multi-view visual consistency and efficient representation learning into dataset distillation. Specifically, biological vision systems process multiple visual observations of the same scene through hierarchical structures in the visual cortex, where complementary information is progressively aligned and integrated into coherent representations. Motivated by this mechanism, our work takes multi-modal medical image feature learning and fusion as the foundation and designs a hierarchical contrastive learning strategy to explicitly model cross-modal consistency.

Furthermore, a key characteristic of biological vision lies in its ability to compress redundant visual inputs into compact and efficient representations through efficient coding mechanisms, enabling long-term retention with minimal redundancy. Inspired by this principle, we reinterpret dataset distillation as a form of visual information compression and incorporate the objectives of cross-modal contrastive learning into the distillation optimization process in a novel manner. This design allows the distilled data to explicitly preserve complementary information across modalities while reducing redundancy, thereby guiding the generation of compact yet informative synthetic samples. While CGMDD is inspired by biological vision and memory systems, we emphasize that our goal is not to faithfully replicate neurobiological processes, but to derive functionally analogous computational principles. Specifically, certain components in our framework are designed as functional abstractions of biological mechanisms. For example, the hierarchical cross-modal contrastive learning scheme reflects the progressive alignment of multi-level representations in the visual cortex, while the dynamic dataset distillation process is inspired by memory consolidation mechanisms that compress redundant experiences into compact representations. These correspondences are not intended to be exact biological implementations. Our framework does not explicitly simulate neural circuitry or temporal dynamics in biological systems. Instead, it adopts biologically motivated design principles to guide the development of efficient and robust learning algorithms. As a result, the proposed approach achieves a balanced trade-off among compression efficiency, diagnostic performance, and privacy protection.

## 3. Methodology

**Figure 2:**
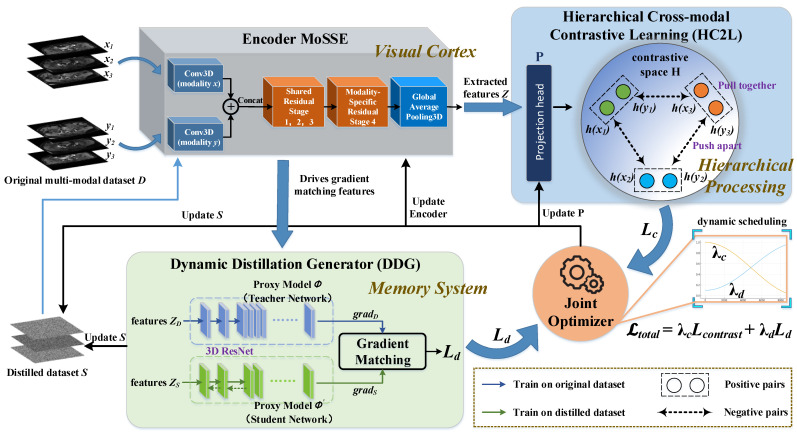
Illustration of the joint optimization framework for the dataset distillation of multi-modal medical images based on a contrast-guided (bio-inspired framework). MoSSE → Visual Cortex: Models multi-modal MRI as multi-view perceptual inputs; HC2L → Hierarchical Processing: Aligns signals across visual channels to form coherent perceptual representations, analogous to multi-level integration in the visual cortex; and DDG → Memory System: Compresses redundant sensory experiences into compact and informative representations, mimicking memory consolidation mechanisms.

This section details the proposed CGMDD framework, which jointly optimizes the cross-modal contrastive learning objective and the dataset distillation process to synthesize a compact yet informative multi-modal medical imaging dataset *S*. By projecting data into a low-dimensional anonymized feature space before distillation, the method both compresses large-scale MRI into lightweight representations and removes patient-identifiable information, enhancing efficiency and security in medical data sharing. The overall framework of our proposed method is illustrated in Figure 2. The framework integrates three key components: the modality-specific and shared encoder (MoSSE) that extracts deep feature representations from original or synthetic multi-modal images; the hierarchical cross-modal contrastive learning (HC2L) module, which explicitly guides the encoder to learn modality-invariant and discriminative representations via contrastive loss; and the dynamic distillation generator (DDG) with teacher–student networks that leverages a gradient matching-based strategy to optimize the synthetic dataset *S*. These three parts work together in a unified optimization process.

### 3.1. Modality-Aware Feature Encoding Strategy

In multi-modal medical image distillation, it is essential to design an encoder architecture that can effectively model both general representations across modalities and modality-specific features. To this end, we adopt the standard 3D ResNet-18 as the encoder backbone, leveraging its capability to process volumetric medical imaging data and capture spatial contextual information. The encoder is further adapted to handle multi-modal inputs through structural modifications, enabling comprehensive modeling of inter-modality commonality and uniqueness, thereby providing a robust foundation for the subsequent contrastive learning and distillation processes.

The architecture of MoSSE is inspired by the hierarchical organization of the human visual cortex, where early layers extract shared low-level features (e.g., edges and textures), while higher-level regions specialize in modality-specific semantic representations.

The encoder MoSSE consists of an initial convolutional layer, a pooling layer, and four residual stages, mapping the input original multi-modal sample x∈D or synthesized multi-modal sample $\tilde{x}\in S$ into the corresponding feature space z=E(x) or $\tilde{z}=E\left(\tilde{x}\right)$. To accommodate multi-modal input, we modify the initial convolutional layer by adjusting the number of input channels to *M* (the number of modalities). Specifically, we introduce modality-specific initial convolutional layers, each handling a single modality. Their outputs are concatenated along the channel dimension and subsequently fed into the shared ResNet body. A partially shared strategy is employed to achieve parameter sharing. The first three residual stages of the encoder capture general low-level and mid-level features and share parameters across all modalities to promote knowledge sharing while reduce the overall parameter count. In contrast, the final residual stage and the global average pooling layer are used to capture high-level semantic features, and independent branches are set for each modality to preserve modality-specific information. This design effectively balances modality invariance and modality specificity, which is crucial for learning discriminative and complementary features in multi-modal medical imaging.

The input to the feature extraction process consists of preprocessed multi-modal image patches with a shape of $C\times D^{\prime }\times H^{\prime }\times W^{\prime }$. The encoder outputs hierarchical feature maps at different levels. For contrastive learning, we use the feature vectors output by the modality-specific branches, which is a 2048-dimensional vector obtained after global average pooling. To facilitate faster convergence and enhance representation quality during training, the shared portion of the encoder is initialized with weights pretrained on LUNA16, a large-scale single-modality medical imaging dataset.

### 3.2. Hierarchical Contrastive Strategy for Cross-Modal Information Fusion

Inspired by cross-sensory integration in biological perception, where the brain aligns signals from different sensory channels to form a coherent perceptual representation, we design a hierarchical cross-modal contrastive learning strategy. This mechanism mimics how biological systems enforce perceptual consistency across modalities through similarity-driven neural responses. Globally, the strategy enforces distributional alignment between synthetic and original data, preserving high-level semantic consistency across modalities. locally, it maximizes the mutual information between features extracted from corresponding anatomical regions across different modalities, thereby promoting joint modeling of anatomical and functional characteristics. This effectively mitigates the heavy reliance on strict voxel-wise registration required by traditional approaches, and makes the method more applicable to real-world multi-modal medical imaging scenarios.

Driven by this motivation, we design a cross-modal contrastive loss $L_{c}$, based on a variant of the InfoNCE loss [60], which has demonstrated robustness in self-supervised learning settings with a large number of negative samples. Unlike conventional contrastive methods, our hierarchical strategy constructs cross-modal positive pairs as well as intra- and inter-modal negative pairs across multiple spatial and semantic scales. Feature embeddings $\left(z^{\left(m\right)},z^{\left(n\right)}\right)$ from different modalities but corresponding to the same spatial location or anatomical region are treated as positive pairs, while embeddings from different locations or different samples are treated as negative pairs. Intuitively, this objective achieves cross-modal feature space alignment by encouraging features from different modalities but the same anatomical location to be close, while pushing apart the feature distance from different structures or different samples. Formally, the loss is defined as:

(1) $$L_{c}\left(z^{\left(m\right)},z^{\left(n\right)}\right)=-\log\frac{\exp\left(\mathrm{sim}\left(z^{\left(m\right)},z^{\left(n\right)}\right)/\tau \right)}{\sum_{k=1}^{N}\exp\left(\mathrm{sim}\left(z^{\left(m\right)},z^{\left(k\right)}\right)/\tau \right)},$$

where $z^{\left(m\right)}$ and $z^{\left(n\right)}$ represent feature vectors from the same spatial location but different modalities *m* and *n*. sim(u,v)=u·v/(∥u∥∥v∥) refers to cosine similarity function, and τ is a temperature hyperparameter. The summation includes one positive pair $\left(z^{\left(m\right)},z^{\left(n\right)}\right)$ and N−1 negative pairs $\left(z^{\left(m\right)},z^{\left(k\right)}\right)$. Negative samples can originate from different spatial locations or modalities within the same image, or from other images within the mini-batch.

Specifically, a multi-modal sample in a mini-batch is denoted as $x_{i}=\left\{x_{i}^{\left(1\right)},x_{i}^{\left(2\right)},\ldots ,x_{i}^{\left(M\right)}\right\}$, consisting of *M* different modalities, where $x_{i}^{\left(m\right)}$ represents the image from the *m*-th modality. The corresponding feature representations extracted by the encoder are denoted as $z_{i}=\left\{z_{i}^{\left(1\right)},z_{i}^{\left(2\right)},\ldots ,z_{i}^{\left(M\right)}\right\}$, where $z_{i}^{\left(m\right)}=E^{\left(m\right)}\left(x_{i}^{\left(m\right)}\right)$, and $E^{\left(m\right)}$ refers to the encoder for modality *m*, which may be shared or partially shared across modalities. For a given modality-specific feature $z_{i}^{\left(m\right)}$, the corresponding cross-modal positive sample is $z_{i}^{\left(n\right)}$, the feature extracted from another modality *n* (n≠m) at the same anatomical location. The negative samples include:

- Inter-instance negatives: All modality features $\left\{z_{j}^{\left(1\right)},z_{j}^{\left(2\right)}\ldots ,z_{j}^{\left(M\right)}\right\}$ from other samples $x_{j}$ (j≠i) within the same mini-batch;
- Intra-instance spatial negatives: Features from different anatomical locations within the same sample $x_{i}$, obtained via patch selection;
- Augmentation negatives: Distorted views of $z_{i}^{\left(m\right)}$ generated through data augmentation such as rotation or noise injection.

Define $L_{contrast}$ as the total InfoNCE loss summed over all modality pairs (m,n). By computing a weighted sum of contrastive losses $L_{c}$ across different modality pairs (m,n) and feature levels, we obtain the final total contrastive loss $L_{contrast}$ as follows:

(2) $$L_{contrast}=-\frac{1}{B\cdot M(M-1)}\sum_{i=1}^{B}\sum_{m=1}^{M}\sum_{n\neq m}\log\frac{\exp(\mathrm{sim}\left(p\left(z_{i}^{\left(m\right)}\right),p\left(z_{i}^{\left(n\right)}\right)\right)/\tau )}{\sum_{k\in N_{i}^{\left(m\right)}}\exp\left(\mathrm{sim}\left(p\left(z_{i}^{\left(m\right)}\right),p\left(z_{k}\right)\right)/\tau \right)},$$

where *B* represents the mini-batch size, and the loss is normalized by averaging over both the batch samples and all modality pairs. This normalization ensures stable optimization and consistent scaling across different batch sizes and modality settings. τ is a temperature hyperparameter that regulates the sensitivity of the model to negative samples. $N_{i}^{\left(m\right)}$ denotes the set of both positive and negative samples associated with the feature $z_{i}^{\left(m\right)}$ from modality *m* of sample $x_{i}$. This set includes one positive sample (i.e., a feature $z_{i}^{\left(n\right)}$ from a different modality *n* at the same anatomical location) and all other negative sample features $z_{k}$. The function p(·) denotes a projection head implemented as a two-layer multilayer perceptron (MLP), which maps the feature *z* to a lower-dimensional latent space of 128 dimensions for contrastive loss computation. This projection facilitates the removal of task-irrelevant information and improves the discriminative quality of the learned representations.

Our hierarchical contrastive strategy primarily constructs negative samples from within the mini-batch, offering a simple yet effective approach. For positive sample pairs, since the input multi-modal images are precisely registered, features corresponding to the same spatial index across modalities naturally form positive pairs. Additionally, to capture multi-scale information, we apply the contrastive loss to feature maps extracted from different stages of the encoder and aggregate them using a weighted sum, enabling multi-level contrastive learning.

### 3.3. Multi-Modal Dataset Distillation Strategy

From a bio-inspired perspective, dataset distillation can be interpreted as a form of memory consolidation, where redundant sensory experiences are compressed into compact and informative representations, similar to how memory systems in the brain encode and remember experiences for long-term storage. This study employs a gradient matching-based dataset distillation strategy. The objective is to optimize the synthetic dataset $S={\left\{\left({\tilde{x}}_{j},{\tilde{y}}_{j}\right)\right\}}_{j=1}^{N_{S}}$ such that the gradients of a randomly initialized downstream model $\phi^{\prime }$, when trained on *S*, closely approximate those of the same architecture ϕ trained on the original dataset $D={\left\{\left(x_{i},y_{i}\right)\right\}}_{i=1}^{N_{D}}$ ($N_{S}\ll N_{D}$). By minimizing the discrepancy between these gradient trajectories, the distilled data can effectively simulate the training dynamics of the original dataset. Ideally, if training on *S* can faithfully replicate the training dynamics observed on *D*, the resulting model trained on *S* should achieve performance comparable to that trained on *D*. To improve efficiency, both ϕ and $\phi^{\prime }$ are instantiated as compact 3D ResNet architectures serving as proxy models. Gradient matching is performed only on randomly sampled subsets of the original dataset and the synthetic dataset, significantly reducing computational overhead.

The optimization objective is to minimize the distillation loss $L_{d}$, which is formally defined as:

(3) $$L_{d}=\sum_{t=0}^{T-1}E_{\theta_{0},D_{t},S_{t}}\left[d(\nabla_{\phi^{\prime }}L_{task}\left(\theta_{t},S\right),\nabla_{\phi }L_{task}\left(\theta_{t},D\right))\right],$$

where $\theta_{t}$ denotes the parameters of the proxy model at iteration *t*. Each iteration starts from randomly initialized parameters $\theta_{0}$, simulating a short training trajectory. Mini-batches $D_{t}$ and $S_{t}$ are sampled from *D* and *S*, respectively. The gradients $\nabla_{\phi }$ and $\nabla_{\phi^{\prime }}$ are computed with respect to the parameters of the proxy models ϕ and $\phi^{\prime }$. The total number of gradient matching iterations is denoted by *T*. $L_{task}$ represents the loss function for the downstream task, which is the Cross Entropy Loss in the case of classification. The function d(·,·) denotes the cosine distance, used to measure the directional discrepancy between two gradient vectors and is defined as follows:

(4) $$d\left(g_{1},g_{2}\right)=1-\frac{g_{1}\cdot g_{2}}{∥g_{1}{∥}_{2}\cdot {∥g_{2}∥}_{2}+\epsilon },$$

where $∥g_{1}{∥}_{2}$ and $∥g_{2}{∥}_{2}$ represent the L2 norms of the vectors, and ϵ is a small constant added for numerical stability.

During the optimization of the synthetic dataset *S*, the pixel values of each synthetic sample ${\tilde{x}}_{j}$ are treated as learnable parameters. They are initialized by sampling from a Gaussian distribution with the same dimensionality as the input images. Specifically, each synthetic sample is initialized as:

(5) $${\tilde{x}}_{j}∼N\left(0,\sigma^{2}I\right),$$

where σ is set to match the empirical variance of the real dataset, and *I* denotes the identity matrix. We also experimented with alternative initialization strategies, including randomly sampled real images and class-wise mean initialization, and observed comparable performance, suggesting that our method does not heavily rely on a specific initialization scheme.

In each outer-loop iteration, the gradient of the distillation loss $L_{d}$ with respect to ${\tilde{x}}_{j}$, denoted as $\nabla_{{\tilde{x}}_{j}}L_{d}$, is computed. The pixel values of ${\tilde{x}}_{j}$ are then updated using the Adam optimizer based on this gradient.

(6) $${\tilde{x}}_{j}\leftarrow {\tilde{x}}_{j}-\eta_{S}\nabla_{{\tilde{x}}_{j}}L_{d},$$

where $\eta_{S}$ denotes the learning rate for optimizing the synthetic data, which is considered a learnable parameter during the distillation process. Additionally, the corresponding synthetic label ${\tilde{y}}_{j}$ is obtained from the predictions of a model trained on the original dataset and remains fixed throughout the optimization.

### 3.4. Contrast-Guided Joint Optimization

The core of CGMDD lies in integrating the contrastive loss $L_{contrast}$ (Equation (2)) into the distillation process, supporting a dual-objective joint optimization guided by contrastive learning. This joint optimization process resembles the co-evolution of perception and memory in biological intelligence systems. By tightly coupling the cross-modal contrastive loss with the dataset distillation loss, the mechanism is designed to simultaneously capture discriminative intra-modal features and complementary inter-modal information. This unified objective not only facilitates the generation of compact synthetic data that preserves critical information but also explicitly enhances cross-modal representation consistency and semantic alignment. To this end, we define the joint optimization objective function $L_{total}$ as follows:

(7) $$L_{total}=\lambda_{d}L_{d}+\lambda_{c}L_{contrast}.$$

The contrastive loss $L_{contrast}$ can be computed using feature representations extracted by the encoder MoSSE from the original dataset *D*, the synthetic dataset *S*, or a combination of both. This ensures that the synthetic data also contribute to learning modality-aligned representations. The hyperparameters $\lambda_{c}$ and $\lambda_{d}$ are used to balance the contrastive and distillation loss terms. To effectively coordinate contrastive learning and distillation, we adopt a cosine annealing schedule to dynamically adjust the weights $\lambda_{c}$ and $\lambda_{d}$ over the course of training. At the early stages, a higher weight $\lambda_{c}$ is assigned to prioritize contrastive learning and facilitate the acquisition of robust cross-modal representations. As training progresses, the weight of the distillation loss $\lambda_{d}$ is gradually increased, placing greater emphasis on gradient matching and knowledge compression. In particular:

(8) $$\lambda_{c}\left(k\right)=\frac{\lambda_{c,\mathrm{init}}}{2}\left(1+\cos(\frac{k}{K_{total}}\pi )\right),$$

(9) $$\lambda_{d}\left(k\right)=\lambda_{d,\mathrm{init}}+\frac{\lambda_{d,\mathrm{final}}-\lambda_{d,\mathrm{init}}}{2}\left(1-\cos(\frac{k}{K_{total}}\pi )\right),$$

where *k* denotes the current outer-loop iteration, and $K_{total}$ is the total number of outer-loop iterations. $\lambda_{c,\mathrm{init}},\lambda_{d,\mathrm{init}}$ and $\lambda_{d,\mathrm{final}}$ represent the predefined initial and final values for the loss weights, respectively.

The joint optimization process can be formulated as a bi-level optimization problem involving an inner loop and an outer loop. In the inner loop, given a fixed synthetic dataset *S*, the downstream model is trained for *T* steps to simulate the learning trajectory, during which the gradient $\nabla_{\phi^{\prime }}L_{task}\left(\theta_{t},S\right)$ is computed. Simultaneously, the corresponding gradient $\nabla_{\phi }L_{task}\left(\theta_{t},D\right)$ is calculated on the original dataset *D*. In the outer loop, both the distillation loss $L_{d}$ (Equation (3)) and the contrastive loss $L_{contrast}$ are evaluated. Based on the total loss $L_{total}$, we perform gradient descent to update the optimizable parameters of the synthetic dataset *S*, including pixel values and generator parameters. The complete procedure of our method is summarized in Algorithm 1.

To ensure stable optimization, we adopt several strategies. First, we normalize the feature representations before contrastive learning, which prevents scale instability across modalities. Second, the gradient matching loss is computed using mini-batch estimates, reducing variance during optimization. In addition, we apply a progressive weighting schedule for the alignment and distillation objectives, which helps avoid optimization conflicts in early training stages. Empirically, we observe that the training process converges smoothly without noticeable oscillations or collapse.
**Algorithm 1** Gradient-matched dataset distillation with multi-modal contrastive learning**Input:** *D*: original multi-modal dataset; *S*: synthetic dataset; ϕ: proxy model; $K_{total}$: total outer iterations; *T*: inner loop steps; $\eta_{S},\eta_{\theta }$: Learning rate; $\lambda_{c,\mathrm{init}}$: multi-modal hyperparameter; $\lambda_{d,\mathrm{init}},\lambda_{d,\mathrm{final}}$: distillation hyperparameter**Output:** $L_{total}$: total loss; *S*: optimized distilled dataset  1:Initialize synthetic dataset S← Gaussian noise randomly, projection head p(·)← two-layer MLP, $\eta_{S}=0.01$, $\eta_{\theta }=1e^{-3}$, $\lambda_{c,\mathrm{init}}=1.0$, $\lambda_{d,\mathrm{init}}=0.1$, $\lambda_{d,\mathrm{final}}=1.0$  2:**for** each outer iteration k=1 to $K_{total}$ **do**  3:    Initialize proxy model parameters $\theta_{t}=\theta_{0}$ randomly  4:    Compute $\lambda_{c}\left(k\right)$ and $\lambda_{d}\left(k\right)$ using cosine annealing  5:    **for** each inner step t=1 to *T* **do**  6:        Sample mini-batch $D_{t}$ from *D* and $S_{t}$ from *S*  7:        Compute gradient w.r.t $\theta_{t}$ and use same $\theta_{t}$ for matching: $grad_{S}=\nabla_{\phi^{\prime }}L_{task}\left(\theta_{t},S_{t}\right)$, $grad_{D}=\nabla_{\phi }L_{task}\left(\theta_{t},D_{t}\right)$  8:        Compute the multi-modal contrastive loss $L_{c}$ using features from $D_{t}$ and $S_{t}$: Equation (1)  9:        Update proxy model parameters:        $\theta_{t}\leftarrow \theta_{t-1}-\eta_{\theta }\nabla_{\theta_{t-1}}L_{task}$10:    **end for**11:    Compute the distillation loss $L_{d}$: Equation (3)12:    Compute the total contrastive loss $L_{contrast}$: Equation (2)13:    Compute the final loss for the distillation process to optimize the synthetic dataset $S_{t}$: Equation (7)14:    Compute gradient w.r.t $S_{t}$ parameters: $\nabla_{S_{t}}L_{total}$15:    Update $S_{t}\leftarrow S_{t}-\eta_{S}\nabla_{S_{t}}L_{total}$16:**end for**17:**return** optimized distilled dataset *S*

## 4. Experiments

### 4.1. Experimental Setup

#### 4.1.1. Dataset

- **MRNet** https://stanfordmlgroup.github.io/competitions/mrnet/ (accessed on 9 January 2019) has 1370 knee MRI scans collected from the Stanford University Medical Center, with 1104 abnormal cases, including 319 ACL tears and 508 meniscal tears. The data consist of three types of images: sagittal T2, coronal T1, and axial PD. These modalities provide complementary anatomical information of the knee, supporting tasks such as abnormality detection and injury classification.
- **JLURM** https://github.com/wjx0818/ (accessed on 27 August 2025) has 32 rectal cancer MRI cases collected from the Radiology Department of Jilin University Sino-Japanese Friendship Hospital, categorized into T2 and T3 stages of rectal cancer. Each category includes axial T2-weighted and contrast-enhanced T1-weighted images (covering arterial, venous, and delayed phases), constituting a rectal cancer MRI dataset.

#### 4.1.2. Implementation Details

In our experiments, we focus primarily on evaluating classification tasks. More complex tasks—such as medical image detection and segmentation—have not yet been explored. This work represents a step-by-step progression, and we plan to investigate these advanced tasks in future research stages. For classification tasks on the MRNet and JLURM datasets, we employ 3D ResNet-18 [61] as the backbone network, which consists of a feature extraction module and a classification head. Within the CGMDD framework, the feature extraction module leverages hierarchical cross-modal contrastive learning (mentioned in Section 3.2), pretrained in an unsupervised manner to achieve multi-modal feature fusion. For MRNet, the feature maps have a shape of (batch size, 256, 256, 256), while for JLURM, the feature fusion module extracts features into a shape of (batch size, 128, 16, 16), with the final synthesized feature map matching the size of the output feature map. Regarding the contrastive strategy, the number of negative samples is set to 4096, the momentum for dynamic memory updating is 0.5, and the temperature parameter for distribution adjustment is set to 0.07. A two-layer MLP (2048 → 1024 → 128) is used as the projection head. During the distillation optimization process, the proxy model also uses 3D ResNet-18. For optimization details, the number of inner-loop gradient matching steps *T* is set to 10, with a learning rate $\eta_{\theta }$ of $1\times 10^{-3}$. The total number of outer-loop iterations $K_{total}$ is set to 10,000. We adopt the Adam optimizer with an initial learning rate $\eta_{S}$ of 0.01. The hyperparameters $\lambda_{c,\mathrm{init}}$ and $\lambda_{d,\mathrm{init}}$ are initialized to 1.0 and 0.1, respectively, with $\lambda_{d,\mathrm{final}}$ set to 1.0 to enable dynamic weight scheduling. The feature fusion module is pretrained for a total of 200 epochs, with a batch size of 32. All experiments were conducted using PyTorch 1.13.0 on a server equipped with two NVIDIA RTX 4090 (24 GB) GPUs.

### 4.2. Effectiveness Analysis of Multi-Modal Feature Fusion

To validate the effectiveness of multi-modal feature fusion, we compare the advantages of our method against the single-modality approach. For a fair evaluation, the same backbone network [61] is employed to perform classification tasks on two multi-modal medical image datasets, MRNet and JLURM. Table 1 presents the classification performance comparison between single-modality and our multi-modal fusion method, using Accuracy (Acc) and Macro-F1 (F1-score averaged across classes) as evaluation metrics, to comprehensively reflect the model’s discriminative ability and class balance. On the JLURM dataset, our method significantly outperforms single-modality models. After fusing images from the three enhanced phases (arterial, venous, and delayed), the accuracy increases from the highest single-modality value of 0.785 to 0.847, and Macro-F1 increases from 0.791 to 0.844, bringing Acc improvements of 22.1% and 8.2% compared to the arterial and venous phases, respectively. In addition, our method also achieves significant performance gains on the MRNet dataset. Compared to single modalities, the accuracy increases from 0.813 to 0.862, and the F1-score rises from 0.821 to 0.857 after multi-modal fusion, with the Acc improvement for Coronal T1 reaching as high as 23.9%.

This results support our central claim that MRI images from different perspectives can express tissue structures more comprehensively after fusion, thereby improving diagnostic performance. The complementary information across modalities works synergistically to enhance feature representations—much like how clinicians rely on integrated insights from multiple imaging sequences to arrive at more precise diagnoses.

### 4.3. Evaluation Under Extreme Dataset Compression

To comprehensively assess the classification performance of our proposed CGMDD method under extreme data compression scenarios, we generated distilled datasets at a 5% compression ratio (CR) on JLURM and MRNet, and conducted comparative experiments against other approaches. All models are evaluated on the corresponding test sets from the original datasets. As presented in Table 2, CGMDD achieves the best performance across both datasets, with accuracy and Macro-F1 scores significantly outperforming baseline methods. Remarkably, even with the distilled dataset being only 5% the size of the original, our method delivers results that closely approach those of the Upper Bound trained on the full dataset.

On the JLURM dataset, CGMDD achieves an accuracy of 0.847 and a Macro-F1 score of 0.844, only 2.5% and 2.1% lower than the Upper Bound (0.872/0.865), respectively. These results substantially surpass those of traditional dataset condensation methods such as DC (0.755/0.748), which is applied in our experiments directly to concatenated multi-channel data, and the kernel-based approach KIP (0.739/0.726). Compared to training with a random subset, CGMDD yields over a 16% improvement. Moreover, it clearly outperforms the two ablation variants: using only cross-modal contrastive pretraining (Only-HC2L) or only multi-modal dataset distillation (Only-DDG), highlighting the critical role of jointly optimizing contrastive learning and gradient matching in enhancing the effectiveness of distilled data. On the MRNet dataset, CGMDD also shows strong performance, reaching 0.862 in accuracy and 0.857 in Macro-F1, only around 2% below the Upper Bound, while outperforming strong baselines DC (0.758/0.751) and KIP (0.746/0.740). It is also worth noting that CGMDD improves accuracy by 15.9% over Only-DDG on MRNet, further confirming the importance of contrastive guidance in the multi-modal distillation process.

To further strengthen the evaluation, we additionally compare CGMDD with several recent multi-modal dataset distillation approaches, including LoRS, EDGE, and PDS, which represent more advanced baselines in this domain. As shown in Table 2, CGMDD consistently outperforms these recent methods across both JLURM and MRNet datasets under the same compression ratio. For example, on the JLURM dataset, CGMDD achieves an accuracy of 0.847, surpassing LoRS (0.819), EDGE (0.830), and PDS (0.837). A similar trend is observed on MRNet, where CGMDD achieves the best performance among all compared methods. Compared with these approaches, which primarily rely on modality fusion or independent distillation strategies, our method explicitly enforces cross-modal alignment during the distillation process through a unified optimization framework. These results validate that incorporating cross-modal alignment into dataset distillation leads to more informative and robust synthetic representations, which is particularly important for preserving complementary information in multi-modal medical imaging scenarios. In summary, CGMDD consistently exhibits robust data compression retention and generalization across different MRI datasets. Even under an extreme 5% compression ratio, it maintains performance close to training on the full dataset, underscoring its potential for application in real-world resource-constrained clinical settings.

**Figure 3:**
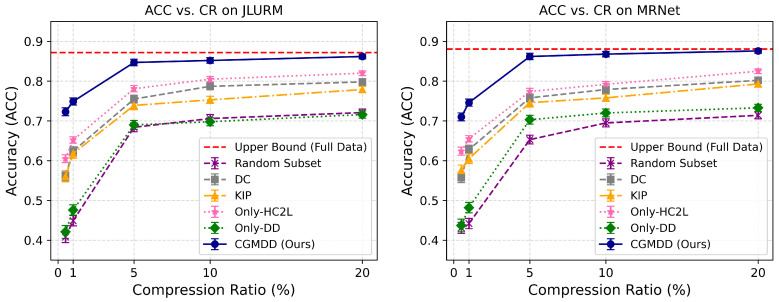
Accuracy curves of JLURM and MRNet datasets under different compression ratios (CR). Error bars represent the standard deviation of three cross-validations.

Furthermore, we evaluate the classification accuracy of different methods under various compression ratio (CR) settings. As illustrated in Figure 3, CGMDD consistently outperforms all comparison methods at every compression level, demonstrating superior distillation capability. Especially at an extremely low compression ratio of 0.5%, CGMDD maintains an accuracy exceeding 0.70, with no significant performance degradation. This represents an improvement of over 10% compared to traditional approaches such as Random Subset, DC, and KIP, as well as the ablation variants. These results indicate that CGMDD can still synthesize highly discriminative and representative samples even under extreme data scarcity, highlighting its strong capacity for knowledge retention and transfer. As the compression ratio increases, the performance of CGMDD rapidly approaches the Upper Bound achieved with full-data training, significantly surpassing other baseline methods.

**Figure 4:**
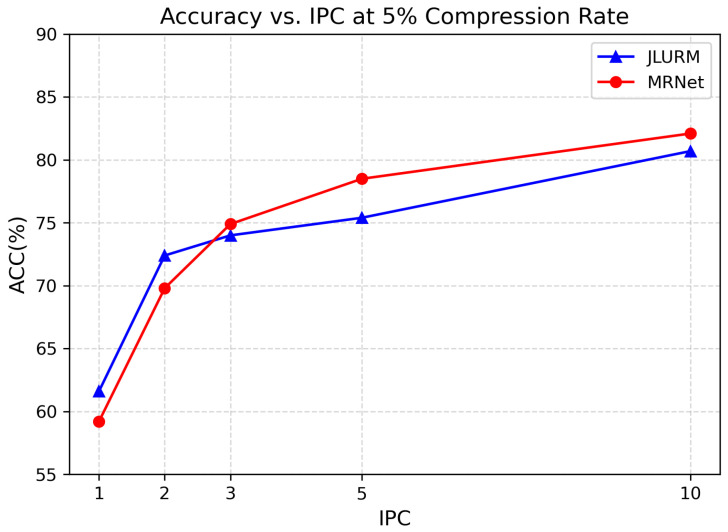
Accuracy achieved with varying amounts of distilled images. IPC (Images Per Class) indicates the number of distilled images per class.

In addition, we observe that once the compression ratio exceeds 5%, the growth of the Acc curve begins to plateau. Taking into account both computational efficiency and storage cost, we regard 5% as the optimal compression ratio in our experiments. As shown in Figure 4, with the compression ratio fixed at 5%, the accuracy of our method improves significantly as more distilled images are gradually added (i.e., as the dataset size increases), demonstrating the positive impact of distilled data quantity on classification performance. In particular, increasing the number of images per class (IPC) from 1 to 2 results in a sharp accuracy boost, suggesting that the model is especially sensitive to distilled images under low-data regimes.

### 4.4. Component-Wise Ablation Analysis

To quantitatively evaluate the contribution of each component in CGMDD, we analyze the performance variations caused by removing individual modules on two datasets under a compression ratio of 5%, as shown in Table 3. Removing the gradient matching distillation strategy leads to the most significant performance drop, with decreases of 39.0% on JLURM and 32.3% on MRNet, indicating that the gradient-based distillation mechanism is the foundation for effective knowledge transfer. Similarly, removing the HC2L module results in substantial degradation (18.5% and 18.4%), highlighting the critical role of contrastive learning in guiding the distillation process to preserve cross-modal consistency. In contrast, removing cross-modal positive pairs reduces the contrastive loss into an intra-modal objective, leading to moderate performance drops (7.6% and 8.8%), which underscores the importance of explicitly modeling cross-modal relationships. Decoupling contrastive learning from distillation (separate training) causes smaller but consistent declines (3.9% and 3.6%), suggesting that joint optimization provides additional benefits by better aligning representation learning with data compression. Although dynamic weight scheduling yields relatively modest gains (0.9% and 0.8%), it consistently outperforms fixed-weight settings, indicating that adapting the optimization focus across training stages is beneficial and validating the effectiveness of the proposed adaptive scheme.

**Figure 5:**
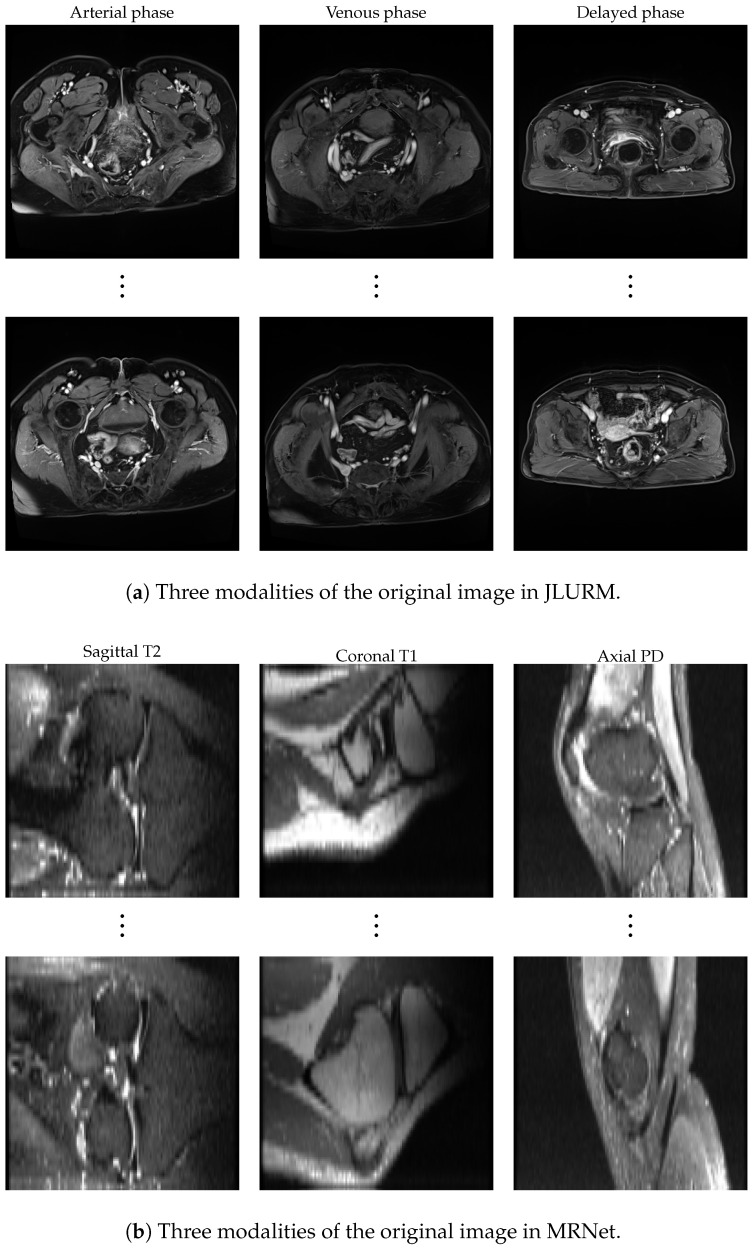
Multi-modal original image display on two datasets.

**Figure 6:**
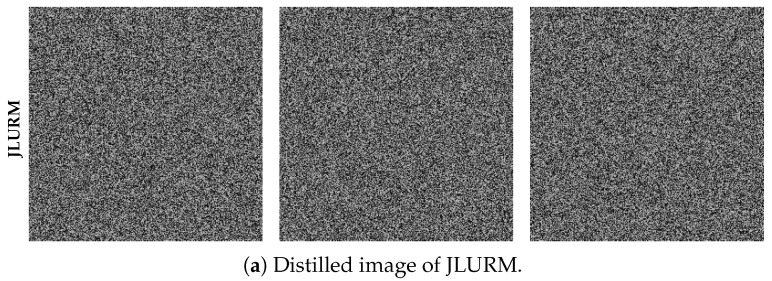

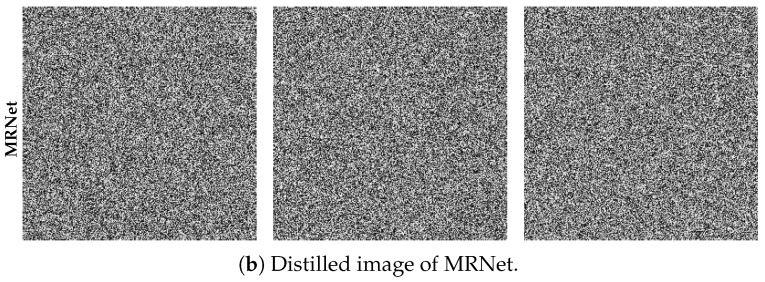
Distilled image display on two datasets. The distilled data exhibit abstract patterns that encode cross-modal structural information rather than pixel-level fidelity.

The ablation study demonstrates that the gradient matching distillation strategy and HC2L are the primary contributors, while the remaining components provide complementary gains, collectively validating the effectiveness of the overall framework design. It is worth emphasizing that, under the guidance of contrastive learning, different modalities do not exhibit mutual interference but instead complement and collaborate with each other, enhancing their respective strengths. Moreover, the distilled synthetic images are generated by optimizing random noise and inherently fuse information from multiple modalities. Consequently, their distribution characteristics and visual similarities differ substantially from the original images. These synthetic samples no longer directly reflect the raw appearance of any single modality but instead carry cross-modal structural information in a more abstract form. Figure 5 shows original samples from different modalities in the JLURM and MRNet datasets, while Figure 6 presents the corresponding distilled data examples. These synthetic samples achieve full visual anonymization, thereby reducing the risk of exposing sensitive patient information. We note that this does not constitute a formal privacy guarantee (e.g., differential privacy), but rather provides a practical way to mitigate privacy risks by avoiding the direct use of real medical data.

### 4.5. The Effectiveness of Label Reduction

Regarding the issue of dataset label acquisition, we investigate the impact of varying label availability by comparing model performance under different label ratios of 30%, 50%, 80%, and 100%. As shown in Table 4, our method maintains strong performance on both datasets, even with limited labels. Especially when only 30% of the labels are available, the classification accuracy using the full distilled dataset still reaches 0.793 and 0.795, achieving performance close to that of the single-modality fully supervised model. This highlights the strong competitiveness of our method under label-scarce conditions and effectively reduces the reliance on extensive annotations.

### 4.6. Hyperparameter Sensitivity Analysis

In our study, two key hyperparameters, $\lambda_{c}$ and $\lambda_{d}$, are introduced in Equation (7) to control the relative contributions of the multi-modal contrastive loss $L_{contrast}$ and the dataset distillation loss $L_{d}$. Given that our approach employs a dynamic weight scheduling strategy, its sensitivity to fixed hyperparameter settings is relatively reduced. Nevertheless, we conducted comparative experiments between dynamic and fixed weight configurations. Specifically, we evaluated the following weight-setting strategies: (1) dynamic weight scheduling via cosine annealing; (2) fixed weights favoring contrastive loss ($\lambda_{c}=1.0$, $\lambda_{d}=0.1$); (3) balanced fixed weights ($\lambda_{c}=0.5$, $\lambda_{d}=0.5$); and (4) fixed weights favoring distillation loss ($\lambda_{c}=0.1$, $\lambda_{d}=1.0$). In the dynamic weight scheduling strategy, higher weight is assigned to the contrastive loss during the early training stages, initialized as $\lambda_{c}=1.0$ and $\lambda_{d}=0.1$, to adequately learn cross-modal consistency representations. As training progresses, the weight gradually shifts toward the distillation loss to promote gradient alignment and knowledge compression.

The experimental results are summarized in Table 5. The dynamic weight scheduling strategy (Group A) achieves the best classification accuracy and Macro-F1 scores on both datasets. In comparison, the model is sensitive to the choice of fixed weights. When a high contrastive loss weight is adopted (Group B1), the model exhibits reasonable generalization capability but insufficient fitting to the distilled data, resulting in performance drops of 6.6% and 9.1% on JLURM and MRNet, respectively. Conversely, overemphasizing the distillation loss (Group B3) leads to faster convergence but inadequate representation learning, which degrades generalization and causes larger performance declines of 10.4% and 13.0%, respectively. The balanced weight setting (Group B2), while providing a compromise between the two objectives, still underperforms the dynamic strategy by 0.8% and 1.1%. These results indicate that improper weighting of the two objectives leads to suboptimal performance, highlighting the model’s sensitivity to fixed hyperparameter choices. In contrast, the dynamic weight scheduling strategy adaptively adjusts the loss weights at different training stages, enabling a more effective balance between representation learning and knowledge compression, thereby mitigating hyperparameter sensitivity and improving overall performance. Moreover, this strategy demonstrates consistent robustness across both datasets and different downstream model architectures. The proposed cosine annealing-based dynamic weight scheduling not only simplifies the process of hyperparameter tuning but also enhances performance in cross-modal learning and distillation compression, confirming the advantages of adaptive optimization strategies in balancing multi-objective losses.

### 4.7. Robustness Under Missing Modalities and Cross-Architecture Generalization

In the modality-missing robustness test, the results in Table 6 show that all models experience performance degradation when certain input modalities are missing. However, models trained with distilled data generated by CGMDD exhibit significantly smaller Acc drops compared to those trained with distilled data from Only-DDG. For instance, in the MRNet dataset, when both ST2 and CT1 modalities are absent, the CGMDD shows a 14.2% decrease in Acc, which is 9.5% lower than the 23.7% drop observed with Only-DDG, demonstrating stronger robustness. This indicates that CGMDD, by incorporating cross-modal contrastive learning, encourages the encoder to learn more modality-invariant feature representations. Even when part of the modality information is missing, the model can still extract sufficient information from the remaining modalities and leverage the learned cross-modal associations to perform a certain degree of information compensation. As a result, the model maintains stable performance under modality-missing conditions.

For dataset distillation methods intended for deployment in real-world application scenarios, a satisfactory distilled dataset should be compatible with diverse downstream model architectures while achieving training performance comparable to the Upper Bound (i.e., fully supervised training). Cross-architecture generalization is therefore a critical metric. We evaluate our distilled dataset on four widely used models: VGG16, MobileNet, ResNet18, and ResNeXt. As shown in Table 7, the distilled dataset generated by the proposed method effectively supports models in attaining both reliable and competitive classification performance, regardless of the model used—ranging from the lightweight MobileNet (only 5.2 M parameters) to the deeper VGG16 (138.4 M parameters). Additionally, training time scales reasonably with model complexity, further demonstrating that our distilled data exhibits robust cross-architecture generalization capability and is adaptable to downstream models of varying complexity.

## 5. Conclusions

In this study, inspired by fundamental principles of the biological vision system, we propose a Contrast-Guided Multi-modal Dataset Distillation (CGMDD) framework for efficient and privacy-preserving learning from multi-modal MRI data. Biological vision systems perceive the same scene through multiple visual observations and process them via hierarchical structures in the visual cortex, where shared low-level features and specialized high-level representations are progressively extracted and integrated. Motivated by this mechanism, we model multi-modal MRI as multi-view visual observations of the same anatomical structure and design the MoSSE that captures both shared and modality-specific representations, analogous to hierarchical processing in the visual cortex. Building upon this representation, we further introduce the HC2L to enforce consistency across modalities, mimicking how the visual system aligns multiple visual inputs into a coherent percept. Meanwhile, the DDG compresses large-scale data into compact synthetic representations, reflecting the efficient coding principle in biological vision, where redundant information is reduced to maximize representational efficiency. Through the joint optimization of HC2L and DDG, CGMDD forms a unified framework that integrates hierarchical perception, multi-view consistency, and efficient coding. This enables effective compression of large-scale multi-modal datasets while preserving critical discriminative information. Experimental results demonstrate that CGMDD maintains strong classification performance under an extreme 5% compression ratio and with only 30% of annotations. These findings further support the effectiveness of incorporating biologically inspired visual mechanisms into dataset distillation, providing a promising direction for developing efficient and interpretable artificial intelligence-based vision systems.

## Figures and Tables

**Table 1:** Experimental results under different modalities. Our approach exploits all three modalities.

Data	Modality	Acc	Macro-F1
JLURM	Arterial phase	0.626	0.638
	Venous phase	0.765	0.770
	Delayed phase	0.785	0.791
	**Multi-modal (Ours)**	**0.847**	**0.844**
MRNet	Sagittal T2	0.813	0.821
	Coronal T1	0.623	0.628
	Axial PD	0.652	0.659
	**Multi-modal (Ours)**	**0.862**	**0.857**

**Table 2:** Comparison of classification performance across different methods on JLURM and MRNet datasets (CR = 5%). “Upper Bound” denotes the performance of the model trained on the full original dataset *D*; “Random Subset” refers to training on a randomly sampled subset $S^{\prime }$ with the same size as the synthetic dataset *S*; “Only-HC2L” uses only cross-modal contrastive pretraining for the encoder, followed by classifier fine-tuning on $S^{\prime }$; and “Only-DDG” applies multi-modal dataset distillation without contrastive guidance (i.e., $\lambda_{c}=0$).

Method	JLURM		MRNet	
	Acc	Macro-F1	Acc	Macro-F1
Upper Bound	0.872	0.865	0.881	0.871
Random Subset	0.684	0.665	0.653	0.634
DC [11]	0.755	0.748	0.758	0.751
KIP [12]	0.739	0.726	0.746	0.740
LoRS [62]	0.819	0.812	0.843	0.833
EDGE [63]	0.830	0.817	0.854	0.848
PDS [64]	0.837	0.831	0.840	0.835
Only-HC2L	0.781	0.763	0.774	0.763
Only-DDG	0.690	0.675	0.703	0.689
**CGMDD (Ours)**	**0.847**	**0.844**	**0.862**	**0.857**

**Table 3:** Quantitative ablation study of CGMDD (CR = 5%). Each variant removes or modifies a specific component to evaluate its individual contribution. The reported “+/−” values denote the relative performance change (%) compared to the full model. The results reveal the relative importance of different components and highlight their complementary roles in the overall framework.

Data	Model Variant	Acc
JLURM	**CGMDD (Full Model)**	**0.847**
	(w/o) HC2L ($\lambda_{c}=0$)	0.690 (−18.5%)
	(w/o) Gradient Matching ($\lambda_{d}=0$)	0.517 (−39.0%)
	(w/o) Joint Optimization (Separate Training)	0.814 (−3.9%)
	(w/o) Cross-modal positive samples	0.783 (−7.6%)
	(w/o) Dynamic Weight Scheduling (Fixed λ)	0.839 (−0.9%)
MRNet	**CGMDD (Full Model)**	**0.862**
	(w/o) HC2L ($\lambda_{c}=0$)	0.703 (−18.4%)
	(w/o) Gradient Matching ($\lambda_{d}=0$)	0.584 (−32.3%)
	(w/o) Joint Optimization (Separate Training)	0.831 (−3.6%)
	(w/o) Cross-modal positive samples	0.786 (−8.8%)
	(w/o) Dynamic Weight Scheduling (Fixed λ)	0.855 (−0.8%)

**Table 4:** Impact of varying label ratios on model performance. IPC denotes the number of synthetic samples per class in the distilled dataset. Notably, performance improves consistently as the label ratio increases, while remaining competitive even at low annotation levels.

Data	JLURM (Label%)				MRNet (Label%)			
IPC	30	50	80	100	30	50	80	100
1	0.569	0.588	0.597	0.616	0.554	0.562	0.585	0.592
2	0.667	0.681	0.703	0.724	0.679	0.687	0.691	0.698
3	0.683	0.705	0.721	0.740	0.712	0.728	0.733	0.749
5	0.708	0.719	0.748	0.754	0.746	0.764	0.780	0.785
10	0.750	0.771	0.782	0.807	0.772	0.793	0.805	0.821
full	0.793	0.803	0.820	0.847	0.795	0.814	0.836	0.862

**Table 5:** Hyperparameter sensitivity analysis of $\lambda_{c}$ and $\lambda_{d}$ under dynamic and fixed weight settings. Different configurations highlight the impact of loss weighting on model performance. Exp. Group represents the experimental group. The results demonstrate that fixed weights lead to noticeable performance variations, while the proposed dynamic scheduling consistently achieves superior and more stable performance across datasets.

Data	$\lambda_{c}$	$\lambda_{d}$	JLURM		MRNet	
Exp. Group			Acc	Macro-F1	Acc	Macro-F1
A (Dynamic)	init	init	**0.847**	**0.844**	**0.862**	**0.857**
B1 (Fixed)	1.0	0.1	0.781	0.769	0.784	0.775
B2 (Fixed)	0.5	0.5	0.839	0.831	0.851	0.845
B3 (Fixed)	0.1	1.0	0.743	0.728	0.732	0.722

**Table 6:** Robustness evaluation under missing modalities.

Data	Missing Modality	Only-DDG Acc Drop	CGMDD Acc Drop	CGMDD Robustness ↑
JLURM	Arterial phase (Ap)	8.5 %	2.7 %	+5.8 %
	Venous phase (Vp)	9.4%	3.1%	+6.3%
	Delayed phase (Dp)	9.9%	5.8%	+4.1%
	Ap + Dp	16.2%	9.2%	+7.0%
MRNet	Sagittal T2 (ST2)	15.7%	10.3%	+5.4%
	Coronal T1 (CT1)	8.6%	3.0%	+5.6%
	Axial PD (APD)	10.4%	3.6%	+6.8%
	ST2 + CT1	23.7%	14.2%	+9.5%

**Table 7:** Generalization performance of the distilled dataset across network architectures.

Data	Indicator	Architecture			
		VGG16	MobileNet	ResNet18	ResNeXt
JLURM	Acc	0.812	0.801	0.847	0.857
	Macro-F1	0.798	0.786	0.844	0.846
	Training time (h)	1.8	0.6	1.1	1.3
	Parameters (M)	138.4	5.2	11.7	25.6
MRNet	Acc	0.825	0.806	0.862	0.866
	Macro-F1	0.815	0.783	0.857	0.854
	Training time (h)	3.5	0.9	1.4	2.2

## Data Availability

The MRNet and JLURM dataset presented in the study are openly available at the following URLs: https://stanfordmlgroup.github.io/competitions/mrnet/ (accessed on 9 January 2019) and https://github.com/wjx0818/ (accessed on 27 August 2025).
